# PAA: an R/bioconductor package for biomarker discovery with protein microarrays

**DOI:** 10.1093/bioinformatics/btw037

**Published:** 2016-01-22

**Authors:** Michael Turewicz, Maike Ahrens, Caroline May, Katrin Marcus, Martin Eisenacher

**Affiliations:** Medizinisches Proteom-Center, Ruhr-University Bochum, Bochum, 44801, Germany

## Abstract

**Summary:** The R/Bioconductor package *Protein Array Analyzer* (*PAA*) facilitates a flexible analysis of protein microarrays for biomarker discovery (esp., ProtoArrays). It provides a complete data analysis workflow including preprocessing and quality control, uni- and multivariate feature selection as well as several different plots and results tables to outline and evaluate the analysis results. As a main feature, *PAA*’s multivariate feature selection methods are based on recursive feature elimination (e.g. SVM-recursive feature elimination, SVM-RFE) with stability ensuring strategies such as ensemble feature selection. This enables *PAA* to detect stable and reliable biomarker candidate panels.

**Availability and implementation:** *PAA* is freely available (BSD 3-clause license) from http://www.bioconductor.org/packages/PAA/.

**Contact:** michael.turewicz@rub.de or martin.eisenacher@rub.de

## 1 Introduction

Protein microarrays (PMs) such as the ProtoArray by Thermo Fisher Scientific, Waltham, MA, USA, are used for autoimmune antibody screening studies, e.g. to discover biomarker candidate panels in human body fluids to discriminate two groups of samples (e.g. ‘diseased’ and ‘controls’). For ProtoArray data analysis the software *Prospector* is often used because it provides the advantageous univariate feature ranking approach minimum M statistic (mMs) (Love, 2007) and a ProtoArray-specific robust linear model normalization (rlm) (Sboner *et al.*, 2009). However, since *Prospector* provides hardly any further functionality for biomarker discovery it is a quite limited tool (Turewicz *et al.*, 2013). Therefore, we have adopted and extended *Prospector*'s key features (mMs, rlm) and implemented *PAA* which provides a complete data analysis pipeline for ProtoArrays and other single color PMs.

## 2 PAA workflow

**Fig. 1:**
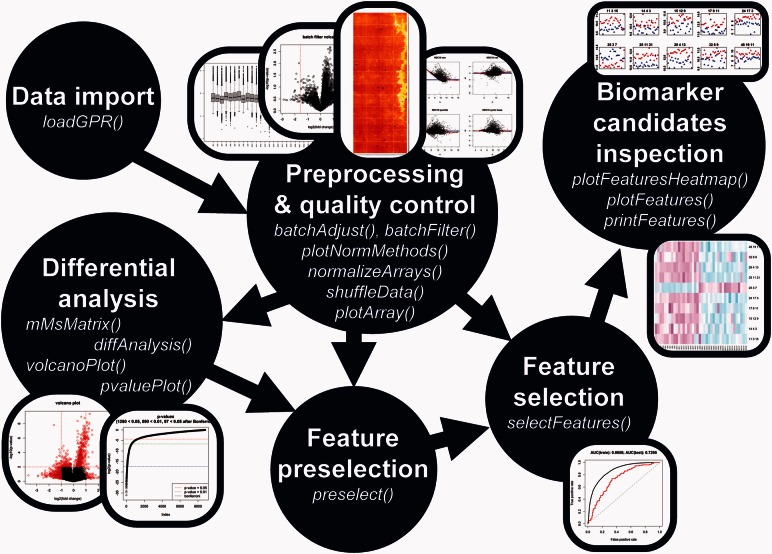
The PAA workflow. The six parts of the *PAA* workflow including their specific function names and plots are shown. Each analysis begins with *‘data import’* and ends with *‘biomarker candidates inspection’*

The adaptable *PAA* workflow consists of six parts (see Fig. 1) which are described in the following subsections.

### 2.1 Data import

*PAA* imports microarray data in gpr file format. Therefore, it provides the function *loadGPR* which imports all needed data into an object of class *EListRaw* (Expression List). To load the desired files and pass metadata not contained in the gpr files (e.g. mapping between sample IDs and gpr files, batch information, clinical data, etc.) a so called targets file has to be created previously and provided to *loadGPR*. In case of ProtoArrays, spot duplicates are condensed by taking the smaller value or taking the mean after data import. Besides ProtoArrays, data of all one color microarrays in gpr file format (e.g. other PMs) can be imported.

### 2.2 Preprocessing and quality control

*PAA* provides several different preprocessing methods to make all PM intensity values inter- and intra-array-wise comparable. E.g. batch effects must be minimized when PMs from different manufacturing lots are compared in large studies (Turewicz *et al.*, 2013). Therefore, *PAA* provides the function *batchFilter* to detect and discard differential features between PM manufacturing lots. Furthermore, the function *batchAdjust* can be used to adjust for known microarray batches. The function *normalizeArrays* provides several different normalization methods. E.g. the ProtoArray-specific rlm approach which uses specific control spots has been reimplemented for *PAA*. Briefly, the model

(1) $$y_{ijkr}=\alpha_{i}+\beta_{j}+\tau_{k}+\epsilon_{ijkr}$$

where $y_{ijkr}$ is the measured spot signal in log2 scale (of array *i*, block *j*, feature *k* and replicate r), $\alpha_{i}$ is the array effect, $\beta_{j}$ is the block effect, $\tau_{k}$ is the actual feature signal and $\epsilon_{ijkr}$ is a random error ($\epsilon_{ijkr}∼N\left(0,\sigma^{2}\right)$) is fitted using robust regression to compute the corrected intensities via ${\hat{y}}_{ijkr}=2ˆ(y_{ijkr}+\alpha_{i}+\beta_{j})$. Other normalization approaches provided by *normalizeArrays* are: cyclic loess, quantile and vsn. To assist in choosing an appropriate normalization method, *PAA* offers two functions: *plotMAPlots* drawing MA plots and *plotNormMethods* drawing box plots visualizing differences before and after normalization. For quality control, the function *plotArray* reconstructs the original spot positions from gpr files to draw a plot mimicking the original scan image and to visualize PMs for which no scan image is available. Then, visual inspection of the spatial intensity pattern can identify strong local tendencies and spatial biases. Moreover, PMs can be inspected after each preprocessing step in order to check the impact of the applied methods.

### 2.3 Differential analysis

*PAA* offers univariate biomarker discovery with fold change and *P*-value calculation via the functions *diffAnalysis*, *pvaluePlot* and *volcanoPlot*.

### 2.4 Biomarker candidate selection

Biomarker candidate selection via feature selection methods is the central task in computational biomarker discovery. Multivariate approaches based on embedded classifier algorithms model feature interdependencies, interact with the classifier and result in more accurate classifications than simpler strategies (Saeys *et al.*, 2007). Hence, *PAA* comes with three recursive feature elimination (RFE) algorithms: (i) a reimplementation of SVM-RFE (Guyon *et al.*, 2002) which utilizes the weights of linear SVMs; (ii) a similar RFE approach using Random Forests (RFs) (Jiang *et al.*, 2004) called RF-RFE; (iii) an interface to RJ-RFE, the RFE method of the *C ++* package *Random Jungle* (RJ) (Schwarz *et al.*, 2010) which is a fast RF reimplementation. All three variants of RFE can be called via the function *selectFeatures* and are embedded in frequency-based feature selection (FFS) (Baek *et al.*, 2009) and ensemble feature selection (EFS) (Abeel *et al.*, 2010) which are strategies that ensure stable and reliable biomarker panels.

### 2.5 Feature preselection

Because RFE embedded in FFS or EFS are computationally expensive multivariate methods for large datasets (e.g. group sizes >30 each) it is often beneficial to reduce the number of variables beforehand. Therefore, *PAA* provides several univariate preselection methods via the function *preselect*. The default method is mMs (implemented in *C ++* to improve run times) which provides a *P*-value based on an urn model (similar approach to the hypergeometric test). Besides mMs, *PAA* provides *t* test- and MRMR-based (Peng *et al.*, 2005) preselection.

### 2.6 Biomarker candidates inspection

*PAA* returns various output for results evaluation. E.g. the plots returned by *pvaluePlot* and *volcanoPlot* visualize differential features from the univariate perspective. ROC curves and results files outlining the classification performance can be returned by *selectFeatures*. After feature selection the resulting biomarker candidate panel can be inspected. Therefore, *PAA* comes with three functions: (i) *plotFeatures* plots the fluorescence intensities of the selected biomarker candidates in group specific colors (one sub-figure per candidate) in order to visualize the differences; (ii) the selected panel and all related protein information can be saved via *printFeatures* into a txt file suitable for analysis with external tools (e.g. annotation); (iii) a heat map of the candidate panel can be plotted by *plotFeaturesHeatmap* as an alternative to *plotFeatures*.

## 3 Conclusion

*PAA* provides a comprehensive toolbox and an adaptable workflow for PM data analysis. It comprises the most important methods of *Prospector* and goes far beyond. Especially the multivariate feature selection based on RFE embedded in FFS or EFS, which is a cutting edge strategy for biomarker discovery, enables *PAA* to identify stable and reliable feature panels. Finally, *PAA* is flexible since the *R/Bioconductor* framework facilitates workflow extension and customization.

## Funding

This work was supported by P.U.R.E., a project of Nordrhein-Westfalen, a federal state of Germany; and de.NBI, a project of the German Federal Ministry of Education and Research (BMBF) [grant number FKZ 031 A 534A].

*Conflict of Interest:* none declared.
